# An Environmental Niche Exploration Tool for Kelp Forest Management

**DOI:** 10.1002/ece3.71459

**Published:** 2025-05-28

**Authors:** Aaron M. Eger, Georgina V. Wood, Jarrett Byrnes

**Affiliations:** ^1^ School of Biological, Earth, and Environmental Sciences University of New South Wales Sydney New South Wales Australia; ^2^ Kelp Forest Alliance Sydney New South Wales Australia; ^3^ Earth Sciences Flinders University Adelaide South Australia Australia; ^4^ Department of Biology University of Massachusetts Boston Boston Massachusetts USA

## Abstract

Sustainably managing kelp forest ecosystems is critical to maintaining marine biodiversity, supporting coastal communities, and meeting global conservation targets such as the Kunming‐Montreal Global Biodiversity Framework's 30 × 30 and Kelp Forest Challenge. Effective kelp forest management frequently depends on selecting environmentally suitable sites that align with species‐specific environmental requirements. This paper introduces a novel kelp forest environmental niche mapping tool that synthesizes the realized environmental niche of 65 kelp species across 25 biophysical factors. Using over 426,000 global observations of kelp and high‐resolution oceanographic datasets, the tool provides quantitative environmental niche data summarized by species and ecoregion. It focuses on key biophysical variables such as temperature, salinity, light, and nutrient availability, offering users practical guidance to identify optimal locations for kelp growth and survival. The tool is accessible via an interactive web application and supports conservation practitioners, policymakers, and researchers by enabling evidence‐based site selection, maximizing conservation success, and informing broader marine ecosystem management. This tool presents a useful advancement in kelp forest management, facilitating global restoration efforts and contributing to the ambitious goal of restoring one million hectares of kelp forest by 2040. Future developments will address qualitative ecological factors and socio‐cultural considerations to enhance its utility.

## Graphical abstract

The kelp niche explorer is a tool which allows users to understand the optimal environmental conditions for kelp forest conservation at the regional level. The tool is adaptable and provides an easy to use graphical dashboard for users to understand kelp forest environmental niches around the world.

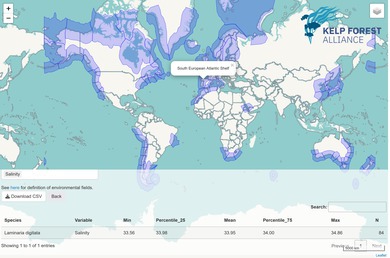

## 1 Introduction

Environmental management practices include several spatially dependent approaches, including traditional protected areas (Watson et al. 2014), Other Effective Area‐Based Conservation Measures (OCEMs), ecological restoration (Gann et al. 2019), ecological engineering (Mitsch and Jørgensen 2003), and nature based solutions (O'Hogain et al. 2018). As the climate crisis intensifies and biodiversity loss accelerates, there is an increasing acknowledgement in scientific, policy, and societal conversations that we need to include this diversity of approaches in management (Reagan 2006; Chazdon et al. 2024). For example, the recent commitment to the Kunming‐Montreal Global Biodiversity Framework's 30 × 30 targets integrates both protection and restoration (Convention on Biological Diversity 2022) while the International Union for the Conservation of Nature champions nature based solutions to protect the ecosystem services needed for in society (Cohen‐Shacham et al. 2016). Implementing many of these management strategies requires sound ecological knowledge of species and ecosystems to ensure success. To meet this need, we must develop efficient tools and frameworks to ensure successful management interventions across various biomes, including marine ecosystems, where restoration has historically lagged behind terrestrial efforts (Abelson et al. 2020; Saunders et al. 2020).

Any management intervention is underpinned by the basic requirement that the target species can persist in the chosen area (Wyant et al. 1995; Matzek et al. 2017). The environmental suitability of a site is critical for the establishment and long‐term survival and health of the species being conserved or managed. As a result, management and conservation projects must consider various biophysical parameters to ensure the target species can thrive in their new environment (Tomlinson et al. 2018). Without carefully matching these factors to the species' ecological needs, any conservation or management effort is likely to fail (Costello and Polasky 2004; Kiesecker et al. 2009; Wood et al. 2024), resulting in wasted resources and potentially further harm to the ecosystem. As such, selecting an environmentally appropriate site is a key step for effective conservation interventions, be they protection, restoration, rewilding, engineering, or otherwise.

Species distribution models (SDMs) or niche models are some of the most popular tools to understand suitable locations for species management. These models use observed species population data and environmental factors to predict where a species might thrive (Elith and Leathwick 2009). These models are built on the assumption that if a species is observed in a particular set of environmental conditions, it can likely thrive in similar conditions elsewhere. SDMs have been applied in a variety of contexts, from wildlife management (Johnson et al. 2012), invasive species control (Srivastava et al. 2019) to the creation of protected areas (Sundblad et al. 2011) and the development of climate change mitigation strategies (Stanton et al. 2012). While these tools have been applied effectively in many ecosystems, they provide a fixed, aggregated output based on the input data. As a result, they do not allow users to adapt the results to their own circumstances, often ignore intraspecific differences in a species' niche, and limit the exploration of key habitat variables, only aggregating habitat indices.

Kelp forests, which are partly made up of marine macroalgae in the order Laminariales, are one of the world's largest and most critical marine ecosystems (Wernberg et al. 2019). Broadly, kelp forests prefer cool waters, high salinity, high wave exposure, high nutrient, high light, and rocky environments (Dayton 1985). Exact conditions vary depending on taxa, population and even specific genotype (Hu and Fraser 2016; Alsuwaiyan et al. 2021). Found along over a third of the world's coastlines (Jayathilake and Costello 2021), the kelp plants in these forests serve as foundation species in temperate marine ecosystems, providing habitat, food, and protection for a wide range of marine life. Kelp forests are also economically important, as they contribute an estimated 500 billion dollars per year in ecosystem services, including carbon sequestration, shoreline protection, and fisheries support (Vasquez et al. 2014; Bennett et al. 2016; Eger et al. 2023). Despite their significance, kelp forests face mounting threats from climate change, pollution, overfishing, and other human activities, leading to widespread declines in many parts of the world (Smale et al. 2019; United Nations Environment Programme 2023).

Contemporary kelp management reflects the broader management practices and includes marine protected areas (Filbee‐Dexter et al. 2024), eco‐engineering on human‐made habitat (Salauddin et al. 2021), or ecological restoration (Eger, Marzinelli, et al. 2022). Of these approaches, restoration is likely the most commonly used worldwide (MPAs are generally not developed specifically for kelp (Arafeh‐Dalmau et al. 2025) and eco‐engineering often focuses more on other species). Generally, kelp forest restoration focuses on remediating physical or chemical stressors (e.g., pollution, Coleman et al. 2008), removing ecological stressors (e.g., grazers, Miller et al. 2024), or reintroducing kelp propagules into the environment (Terawaki et al. 2003; Layton et al. 2021). Ecological engineering in kelp management works to grow kelp in new areas such as the open ocean (Stekoll et al. 2025) or on artificial reefs (Eger, Aguirre, et al. 2024).

There is still considerable progress to be made to conserve the world's kelp forests, including increasing the area under restoration and protection. To date, approximately 19,000 ha of kelp forest habitat have been restored worldwide and 16% of the biome is under some form of protected area, with just 2% in a highly protected area (Eger, Eddy, et al. 2024). These values are just a fraction of what is needed to meet the ambitious targets set by the Kelp Forest Challenge, which calls for the restoring 1 million hectares and protecting 3 million (30% of mapped kelp) hectares of kelp forests by 2040 (Eger, Aguirre, et al. 2024). Achieving this goal will require a massive scaling‐up of conservation efforts, with careful attention to selecting suitable sites. In addition to a number of suitability models for present and future kelp forest habitat (Gorman et al. 2013; Martínez et al. 2018; Sudo et al. 2020; Goldsmit et al. 2021; Assis et al. 2024; Gonzalez‐Aragon et al. 2024), there are several useful tools to aid in kelp forest management (Gleason et al. 2021; Eger, Layton, et al. 2022; Bell et al. 2023; Wood et al. 2024). These tools offer general guidance for kelp forest restoration and region‐specific recommendations for areas that are currently or potentially suitable for kelp forests (Eger, Layton, et al. 2022; Giraldo‐Ospina et al. 2024; Gonzalez‐Aragon et al. 2024).

The existing habitat models, site selection tools, and general practice guidance for kelp forest management are informative but are not designed to guide in‐water efforts. Existing outputs focus on a single species (Giraldo‐Ospina et al. 2024; Gonzalez‐Aragon et al. 2024), a particular region (Martínez et al. 2018; Franco et al. 2018; Goldsmit et al. 2021), or do not provide quantitative values to help guide selecting new sites for restoration (Eger, Layton, et al. 2022). None of the existing habitat models are truly customizable and allow users to explore a range of scenarios, conditions, or geographies. Still, there is a need to inform kelp conservation efforts as the field is growing. To successfully guide future management work, we must understand the existing “realized niche” of specific kelp populations. That is, the specific observed environmental conditions under which kelp populations might grow and reproduce. Granted, there is a wealth of information about the general preferences for kelp forests (e.g., water temperature, nutrient availability, light conditions, and substrate type), but there is no global, consolidated information that accounts for intraspecific variation.

This paper introduces a novel, environmental kelp forest site selection tool that summarizes the realized niche for 65 kelp species across 25 key biophysical factors. The tool integrates multiple environmental variables, including temperature, nutrient availability, light levels, oceanographic conditions, and substrate type, to provide an easily accessible source of information for the “environmental niche” for kelp species within a bioregional context. These data are uniquely summarized by kelp species and ecoregion and provide guidance for practitioners, policymakers, and conservationists about the realized environmental niche values of these species. The tool is made accessible through a user‐friendly web portal, which allows users to access the relevant information for their local context. It then provides a list of relevant databases where users can input this into a site selection framework, thus offering a valuable resource for practitioners seeking to ensure the long‐term suitability of kelp restoration projects.

## 2 Methods

### 2.1 Global Biodiversity Information Facility

We used ~426,000 observations of global kelp forests (filtered by the order Laminariales) from the Global Biodiversity Information Facility (GBIF.org, accessed November 23, 2023) to build profiles of the environmental conditions that kelp species have been observed living in. These data were available for 65 species from 26 genera and were collected between 1821 and 2019. As a result, they reflect the historical realized niche of kelp species in an ecoregion and do not represent species or populations that may be undergoing adaptation to novel or changing environmental conditions. We acknowledge the potential mismatches in dates between the kelp observational data and the environmental data, particularly as sea temperatures are changing (Aral and Guan 2016). Nevertheless, we believe the data describe informative, large‐scale patterns that reflect the evolution over hundreds of years. We are also not aware of any regional extinctions among the species we included (i.e., the environmental conditions are completely unfit for growth and survival). Last, to improve data quality, we filtered the data to only include species‐ecoregion combinations with more than 10 observations and removed any land‐based observations.

### 2.2 World Ocean Layers

We used the globally distributed, consolidated, and freely available Global Marine Environment Datasets (GMED; Basher et al. 2018) to provide the relevant information on the physical, chemical, nutrient, past, and future conditions in the ocean. These 16 data categories were available at resolutions ranging from 30 arc‐seconds to 75 arc‐minutes (Table 1) and were based on observations from 1874 to 2009 (mean start = 1944, mean finish = 2004). We chose the following data categories because they are the key biophysical components of the kelp niche: temperature, salinity, depth, aspect, wave height, current, radiation, chlorophyll‐a, pH, and nutrients (Steneck et al. 2002; Assis et al. 2018; Martínez et al. 2018).

**TABLE 1:** Metadata for the biophysical environmental variables used in this study.

Variable type	Layer	File name code	Description	Unit	Type	Instrument/institute	Original spatial resolution	Temporal range	Derivatives	Source	Primary data source	URL
Chemical	Chlorophyll‐a	bo_chla_x	Chlorophyll A concentration indicates the concentration of photosynthetic pigment chlorophyll A (the most common “green” chlorophyll) in oceans. Please note that in shallow water these values may reflect any kind of autotrophic biomass	mg/m^3^	Monthly climatology	Remote sensing: Aqua‐MODIS	5 arcmin (9.2 km)	2002–2009	Mean, minimum, maximum, range	Bio‐oracle	Feldman and McClain (2010)	http://oceancolor.gsfc.nasa.gov/
Chemical	Chlorophyll‐a	kg_chla_x	Chlorophyll‐a concentration data consists of satellite measurements of global and regional ocean color data	mg/m^3^	Annual climatology	Remote sensing: SEAWiFS/OrbView‐2	~5 arcmin (9 km)	1997–2006	Max, mean, summer max, winter max	KGS mapper	Feldman and McClain (2006)	http://oceancolor.gsfc.nasa.gov/SeaWIFS/
Chemical	Chlorophyll‐a (primary productivity)	aq_primprod	Proportion of annual primary production in a cell. See reference for details about the productivity calculation methods	mgC·m‐^2^/day/cell	Annual climatology	Remote sensing: SeaWiFS	~5 arcmin (9 km)	—	Mean	Aquamaps HCAF v4	Bouvet et al. (2002), Hoepffner et al. (1999), Longhurst et al. (1995)	http://www.aquamaps.org/download/main.php, http://www.seaaroundus.org/doc/saup_manual.htm#3
Chemical	pH	bo_ph	Measure of acidity in the ocean surface	—	In situ measure: WOD 2009	Standard level data: Surface ocean station data (OSD); high‐resolution conductivity‐temperature‐depth (CTD)	1° × 1°	1910–2007	Mean	Bio‐oracle	Boyer et al. (2002)	http://www.nodc.noaa.gov/
Nutrients	Dissolved oxygen	bo_o2dis	Dissolved oxygen concentration [O_2_] in the surface	mL/L	In situ measure: WOD 2009	Standard level data: Surface ocean station data (OSD); high‐resolution conductivity‐temperature‐depth (CTD)	1° × 1°	1898–2009	Mean	Bio‐oracle	Boyer et al. (2002)	http://www.nodc.noaa.gov/
Nutrients	Dissolved oxygen	kg_s_o2saturate	Amount of dissolved oxygen as a percentage of maximum potential oxygen amount that could be present for the given temperature and salinity at standard amospheric pressure (760 mmHg) (i.e., sea level)	mL/L	Annual climatology	World Ocean Atlas 2001	0.5° × 0.5°	1874–2000	Mean (surface)	KGS mapper	Conkright et al. (2002)	http://www.nodc.noaa.gov/OC5/WOA01/pr_woa01.html
Nutrients	Nitrate	bo_nitrate	This surface layer contains both [NO_3_] and [NO_3_ + NO_2_] data. By this we mean chemically reactive dissolved inorganic nitrate and nitrate or nitrite	μmol/L	In situ measure: WOD 2009	Standard level data: Surface ocean station data (OSD)	1° × 1°	1922–1986	Mean	Bio‐oracle	Boyer et al. (2002), NOAA/NGDC Paleoclimatology Program, Boulder CO, USA	http://www.ncdc.noaa.gov/data‐access/paleoclimatology‐data
Nutrients	Phosphate	kg_phosphate/kg_b_phosp	Phosphorous concentration	μmol/L	Annual climatology	World Ocean Atlas 2001	0.5° × 0.5°	1874–2000	Mean (surface, bottom)	KGS mapper	Saving (2006)	http://hercules.kgs.ku.edu/website/specimen_mapper/data/bott_mean06_1.htm
Physical	Aspect (East–West)	msp_aspect_ew	East/West Aspect of seafloor (sin(aspect in radians))	Radians × 100	—	Interpolation	5 arcmin (9.2 km)	—	—	MARSPEC	Becker et al. (2009); Sbrocco and Barber (2013)	http://www.marspec.org/
Physical	Photosynthetically active	bo_parmean	Photosynthetically Active Radiation (PAR) indicates the quantum energy flux from the Sun (in the spectral range 400–700 nm) reaching the ocean surface	Einstein/m^2^/day	Monthly climatology	Temperature‐depth (CTD) (surface)	5 arcmin (9.2 km)	1997–2009	Mean	Bio‐oracle	Feldman and McClain (2010)	http://oceancolor.gsfc.nasa.gov/
Physical	Salinity	bo_salinity	Salinity indicates the dissolved salt content in the ocean surface	PSS	In situ measure: WOD 2009	Standard level data: Surface ocean station data (OSD); high‐resolution conductivity‐temperature‐depth (CTD)	1° × 1°	1961–2009	IDW interpolation	Bio‐oracle	Boyer et al. (2002)	http://www.nodc.noaa.gov/
Physical	Surface current	ecco2_uv_surface_current	Zonal velocity (UVEL), meridional velocity (VVEL)	m/s	Monthly climatology	NASA ECCO2 modeling, analysis, and prediction (MAP) project	0.25° × 0.25°	—	Mean (surface)	NASA ECCO2 modeling	NASA JPL laboratory	http://ecco2.jpl.nasa.gov/
Physical	Temperature	bo_sst_x	Sea surface temperature is the temperature of the water at the ocean surface. This parameter indicates the temperature of the topmost meter of the ocean water column	°C	Monthly climatology	Remote sensing: Aqua‐MODIS	5 arcmin (9.2 km)	2002–2009	Mean, minimum, maximum, range	Bio‐oracle	Feldman and McClain (2010)	http://oceancolor.gsfc.nasa.gov/
Physical	Tide average	kg_tide_average	Tides, avg. of maximum amplitude. These tide model results are from a global 1/4° tide model which assimilated tide estimates derived from the TOPEX/Poseidon altimeter	m	Annual climatology	University of Colorado, Boulder	0.25° × 0.25°	—	Mean	KGS mapper	Stewart (2000)	http://instaar.colorado.edu/~scotts/thesis.pdf
Physical	Waveheight	aq_waveheight	Height of waves in scaled discrete classes as provided by the Original LOICZ Database, for all coastal and oceanic cells	m	—	—	0.5° × 0.5°	—	Mean	Aquamaps HCAF v4	KGS	http://www.aquamaps.org/download/main.php
Physical	Windspeed	kg_wind_speed	Yearly variations of the surface marine atmosphere over the global oceans	m/s	Annual climatology	International Research Institute for Climate Prediction (IRI)	0.5° × 0.5°	1945–1989	Mean	KGS mapper	Da Silva et al. (1994)	http://ingrid.ldgo.columbia.edu/SOURCES/.DASILVA/.SMD94/.climatology/.w3

### 2.3 Extract and Summarize by Ecoregion

We focused on describing the observed environmental values for the kelp species included in our dataset. We did not model habitat suitability, as various habitat suitability modeling approaches often obscure the relationship between individual environmental variables and the species' presence–absence by creating composite indexes. Creating uni‐ or multivariate models of the species was also problematic given the presence‐only nature of the data and associated difficulties with interpreting probability distributions. We therefore collected data on the range of environmental conditions in which kelp was observed and summarized that information for easy interpretation.

We used the extract by location function in QGIS to obtain the corresponding environmental niche data for every one of the 426,000 kelp observations. Because species often exhibit regional niche variation as a result of variation in genetic and phenotypic diversity (Wood et al. 2024), we opted to summarize the niches by the Marine Ecoregions of the World (Spalding et al. 2007). These ecoregions group geographies together based on similar environmental conditions, and species found in the same ecoregion are more likely to have a more similar environmental niche than those found in a different ecoregions (Smith et al. 2020). Admittedly, some ecoregions are quite large and contain a range of environmental conditions. Nonetheless, we believe they maintain a fine balance of providing global information while focusing in on regional and local areas. Finally, we group data by species (*n* = 65) and ecoregion (*n* = 67) and used the summarize function in the *dplyr* package (Wickham et al. 2023) in R to calculate the summary statistics about the different values found for each species in each ecoregion.

We made the simplifying assumption that a species' niche space has a bell curve, with an optimal value in the middle, less preferable values out towards the tails, followed by intolerable values beyond the observed limits (Citores et al. 2020). While simplified, this distribution is reflected in the real‐world niche of many species (Brown 1984; Chen et al. 2024). To reflect these assumptions and help guide the site selection process, we used dplyr in R (Wickham et al. 2023) to calculate the minimum value, 25th percentile, mean value, 75th percentile, and maximum value for each variable for each ecoregion‐species pairing. These values also allow users to explore a range of values and not rely solely on the average.

### 2.4 Shiny App

We developed an interactive, map‐based interface shiny app in R (R Core Team 2013) to streamline user engagement with bioregional biodiversity data (https://www.kelpee.info). Users begin by selecting bioregions of interest from a global map, after which they receive a tailored list of relevant species occurring in those areas. Through intuitive menus, users can then choose up to 25 environmental variables to access summarized data values. To facilitate modeling and enhance data sharing, the interface includes a feature enabling users to view these results in tabular form and download customized csv files containing the selected data along with comprehensive metadata.

## 3 Results and Discussion

**FIGURE 1:**
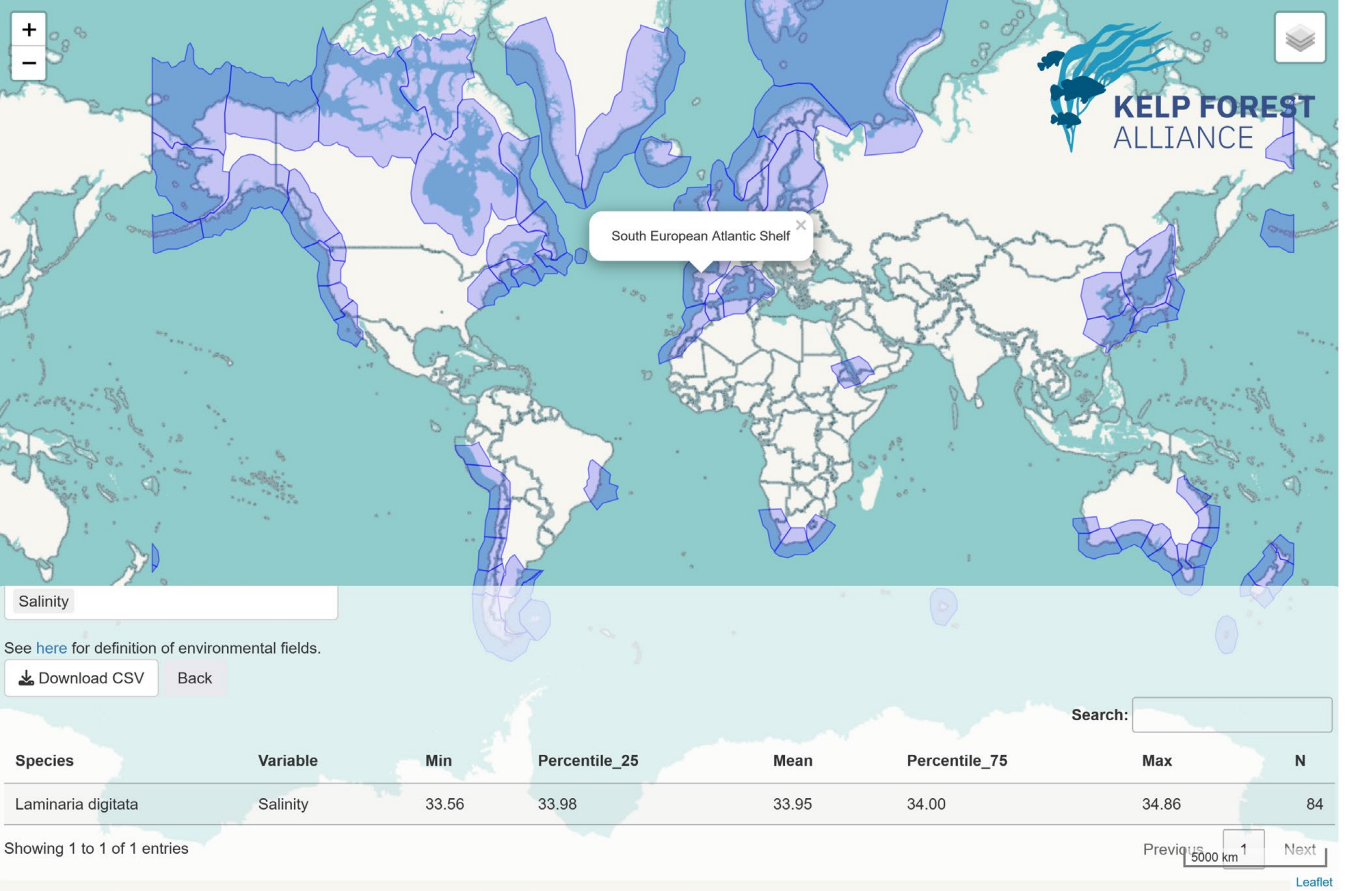
Example table of a species–ecoregion and selected data.

We produced summary information for the 25 ocean variables for 282 species‐ecoregion combinations around the world (Figure 1, Table S1).

**FIGURE 2:**
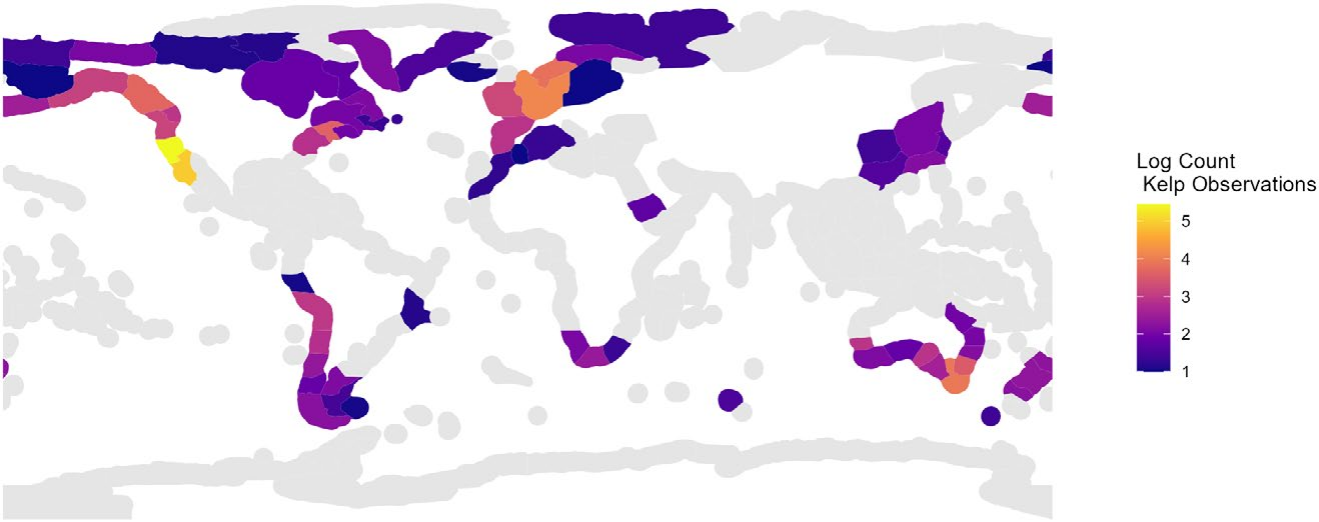
Distribution of the number (logged) of kelp presence records by ecoregion.

There was substantial geographic bias in the available kelp distribution data. Together, the ecoregions Northern California and Southern California Bight contained over 85% of the kelp records (Figure 2).

**FIGURE 3:**
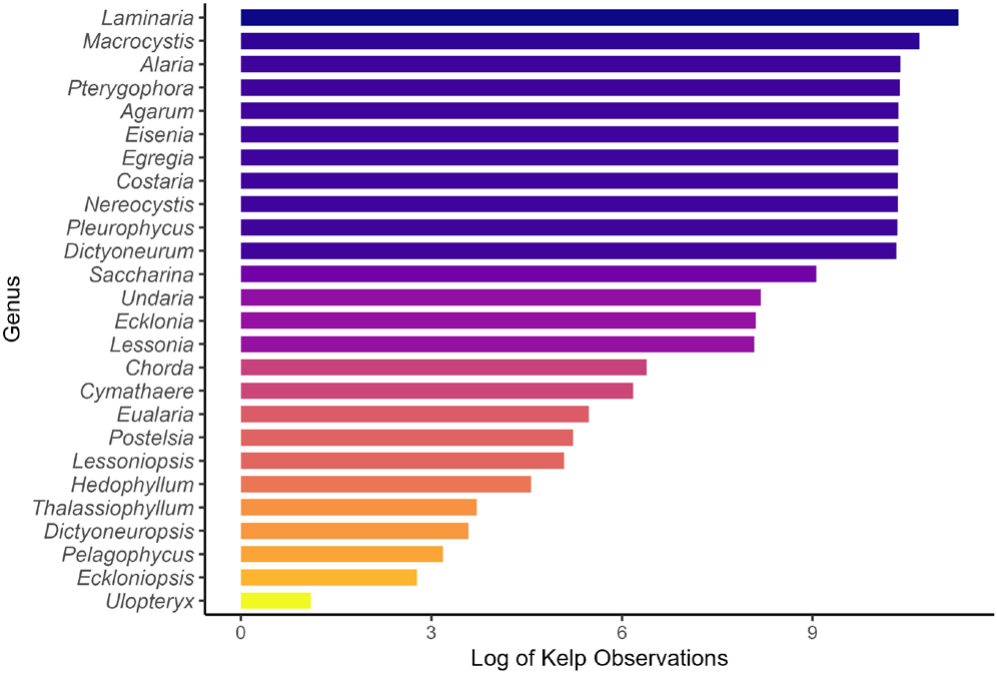
Distribution of the (logged) number of observed kelp presence records by genus.

Areas in gray had no observations. The kelp records were more evenly distributed as 20 species had over 1000 observations and 37 had over 100 observations (Figure 3).

### 3.1 Short User Guidance

These data describe the range and most observed values for 25 environmental factors in each ecoregion. This information can be used to inform different activities related to kelp forest management and research. Users can apply this tool by assessing the environmental requirements of their target species in a given ecoregion. First, they may wish to evaluate the minimum and maximum niche values and ensure that they are not trying to restore a species outside of its regional environmental niche and thus increasing the probability of the species not re‐establishing.

Because species exhibit a bell‐shaped response to environmental conditions (Roughgarden 1974; Antão et al. 2022), users may wish to identify the “optimal” environmental conditions for kelp survival by looking at the ecoregion mean, 25th, and 75th percentiles. Users should attempt to find environmental conditions that range between those three numbers, with a preference for values near the mean.

Users may also use these data to identify regions which have optimal conditions for kelp forest growth and survival. As a result, the tool could help managers and industry proponents summarize optimum conditions for kelp habitat and use this to identify or prioritize the best locations to place marine protected areas or kelp farms. Additionally, where there is local environmental data, but no kelp survey data, users can filter by the specified range of biophysical requirements to find suitable areas for kelp growth. Lastly, the niche breadths presented here can be used to create or test scientific hypotheses about environmental tolerances in field or laboratory experiments (e.g., thermal tolerance tests).

**FIGURE 4:**
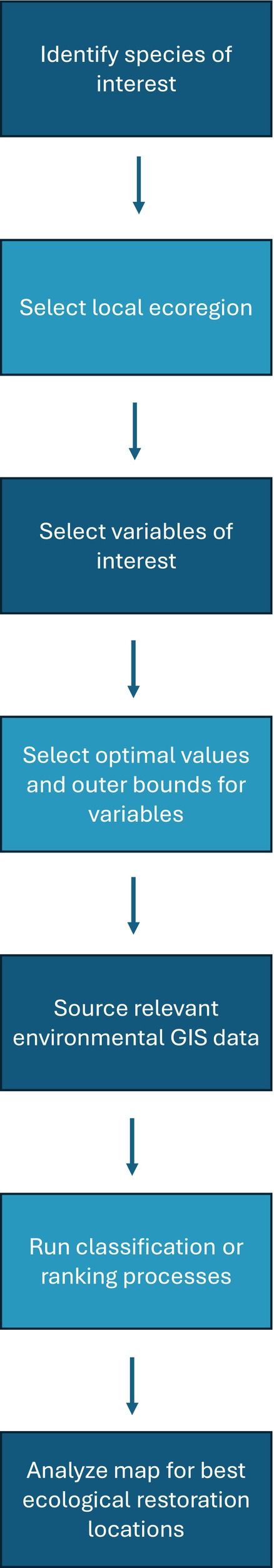
Workflow diagram of how to practically use the application.

Here, we provide a short instructional section to help guide users in accessing the relevant information on the web‐based shiny application (Figure 4).

For example, say a user is interested in restoring *Ecklonia radiata* in Northeastern New Zealand. To determine which sites might be the most environmentally suitable for kelp forests, users load the shiny app, find the region they are interested in working in, and click on the ecoregion on the map. In this instance, the ecoregion *Northeastern New Zealand* was chosen. Once the ecoregion is selected, the user may select the species of interest from the drop‐down menu and identify the environmental variables they wish to look up data for in the second drop‐down menu. There is no limit to the number of variables a user may select. The user is then presented with the relevant summary statistics for each environmental variable in the shiny app. Alternatively, these data may then be downloaded as a csv to be saved or used in other formats.

Once equipped with the relevant statistics, the user must select the desirable values and source geospatial environmental data to aid in selecting suitable sites. One suggestion is to use the values between the 25th and 75th percentiles as the desired range of values. A second is to exclude areas with values outside of the maxima and minima. The user should consider what environmental data they have available, ideally that is data that is locally collected and relevant. National and regional resource agencies often maintain the relevant data layers at higher resolutions. If the user cannot access higher resolution spatial data, they may use the global dataset used to generate these results (Basher et al. 2018), the references for which are provided in the shiny app.

With the geospatial data in hand, the user can find areas with an intersection of the desired and relevant environmental niche value via a relatively simple geospatial analysis (e.g., QGIS, ArcGIS, R). For every environmental layer, the user will need to classify the values as more or less desirable. A simple approach would be to use the raster calculator in ArcGIS or QGIS or the calc function in the R package *raster* (Hijmans et al. 2013) or the app function in the *terra* package (Hijmans et al. 2022), to classify every value between the 25th and 75th percentiles for an environmental variable as a 1 and everything outside of that as a 0. Next, the classified layers can be added together. The resulting raster is thus a map where the most desired areas have the highest values. Users may also wish to weight variables based on their importance by giving them higher scores (e.g., 2s or 3s) or rank values closer to the mean as more desirable (also by giving them higher values) or use the maximum and minimum values as the signposts. The resulting areas with the highest scores are then those that are most environmentally suitable for kelp forest populations in that area. Users may also use zonal statistics (ESRI 2024) to find out the most suitable regions for kelp forest populations.

### 3.2 Additional Considerations for Site Selection

A population is inherently constrained by its environmental niche, but there are many other social, logistical, and cultural factors for selecting conservation sites (Meli et al. 2014). Therefore, while this tool helps users find the most environmentally suited locations, users must also consider the social license to for conservation work (Kendal and Ford 2018), other uses of the marine space (Lester et al. 2020), Indigenous and Traditional views on marine management (Uprety et al. 2012), the feasibility of accessing a site (Piccolo et al. 2024), local knowledge about site suitability (Fremout et al. 2021), ecological factors (e.g., presence of sea urchins) (Miller et al. 2022), and legal constraints (e.g., permitting or zoning) (Hamilton et al. 2020; Bell‐James et al. 2023).

This work focuses on the quantitative biophysical elements of a species' environmental niche and does not present information on the qualitative or ecological components. Future work should conduct a literature review to identify other known components of a species' niche. These factors may include, but are not limited to, substrate type, preferable outplanting season, positive and negative ecological interactions (e.g., herbivory), dispersal distance, or life span.

These data and the corresponding summaries may be easily updated and merged with any new information for restoration, conservation, or kelp farming site selection that becomes available.

## 4 Conclusion

This paper introduces a novel kelp forest management tool that synthesizes the realized niche of 65 kelp species across multiple biophysical parameters. By integrating data on oceanographic conditions, light, nutrient availability, and substrate type, this tool provides a robust resource to guide kelp management globally. It offers a improvements over existing models by allowing users to consider a range of scenarios and is easily accessible to a wide range of users, including conservation practitioners, policymakers, and conservationists. As the urgency to manage marine ecosystems intensifies, this tool will be critical in maximizing the success of future kelp forest conservation efforts. While this tool addresses the biophysical aspects of site selection, further work is needed to consider qualitative ecological factors and socio‐cultural considerations that influence management. By continually updating and refining the tool as new data become available, we can more effectively scale up restoration efforts to meet global biodiversity targets and contribute meaningfully to the conservation of kelp forests worldwide.

## Author Contributions

**Aaron M. Eger:** conceptualization (lead), data curation (lead), formal analysis (lead), funding acquisition (lead), methodology (equal), project administration (lead), writing – original draft (lead), writing – review and editing (equal). **Georgina V. Wood:** conceptualization (supporting), data curation (supporting), formal analysis (supporting), software (lead), writing – original draft (supporting), writing – review and editing (equal). **Jarrett Byrnes:** conceptualization (equal), funding acquisition (lead), project administration (supporting), software (supporting), writing – original draft (supporting), writing – review and editing (equal).

## Conflicts of Interest

The authors declare no conflicts of interest.

## Supporting information


**Table S1.** Summary statistics of environmental variables by species and ecoregion.

## Data Availability

All data used in creating this tool are referenced throughout the document and the raw data are available at the Open Science Framework repository—https://osf.io/ewsk5/.
